# Evaluating the Effectiveness of Home IV Therapy Administration Modalities in Homebound Older Adults

**DOI:** 10.1093/geroni/igaf122.3928

**Published:** 2025-12-31

**Authors:** Kimberly Oakman, Tammy Sanders, Laura Medlin, Sandra Sanchez-Reilly, Hanh Trinh, Valerie Escamilla, Michael Mader

**Affiliations:** Veterans Administration, San Antonio, Texas, United States; South Texas Veterans Health Care System, San Antonio, Texas, United States; South Texas Veterans Health Care System, San Antonio, Texas, United States; South Texas Veterans Health Care System and the University of Texas Health Science Center at San Antonio, San Antonio, Texas, United States; Veterans Health Administration, Veterans Health Administration, Texas, United States; South Texas Veterans Health Care System and UT Health San Antonio, San Antonio, Texas, United States; South Texas Veterans Health Care System, San Antonio, Texas, United States

## Abstract

**Background:**

Over 80% of older adults (OA) suffer from one or more chronic conditions which affect their ability to cope with injuries or disease exacerbations. When acutely ill, OA usually require intravenous medication administration (IVMA), which traditionally has been done when hospitalized, exposing them to increased adverse events. Home-care-programs have increased their scope-of-practice to allow home-IVMA with >85% goal that medication-enters-the-body (METB). Administration methodology comparisons between home infusions (HI) and intravenous-push-administration (IVP-100% METB) have not been studied.

**Objective:**

To compare the IVMA effectiveness using HI vs. IVP in a population of OA receiving IV-treatment at home.

**Methods:**

Using three infusion companies and guaranteeing nurses-led visits, first-part-of-study collected one-year detailed data on IVMA methodology/complications via HI. Subsequently, IVP was encouraged (vs. HI) whenever recommended by pharmacy. One-year data on recommendation follow-up/complications/satisfaction was analyzed.

**Results:**

Overall, 67% were>65, 95%male, age=43-87. First-part-of-study: N = 15, 60% had>10 IVMA episodes using PICC/midline/porta-cath. Only 53% participants met >85% METB goal with standard HI. Second-part-of-study: N = 154, 28% IVMA were recommended to IVP, those subjects receiving 100% METB. When comparing the pre-and post-groups, complication rate was 10% for both, High satisfaction rates were found among the IVP group.

**Conclusions:**

On this preliminary study and when recommended by pharmacy, IVP was shown to be an effective IVMA method as demonstrated by 100%METB without higher complications compared to HI and indicated high patient satisfaction. Further research is needed to evaluate effectiveness of IVP vs. HI among OA, considering nurse manpower, cost-effectiveness (tubing/supplies/pump, infusion preparation, etc.), time-to-completion, METB rates, complications and patient-satisfaction.

